# Management of manifesting *FOXRED1* carriers is complex

**DOI:** 10.1016/j.ymgmr.2019.100463

**Published:** 2019-02-23

**Authors:** Josef Finsterer, Sinda Zarrouk-Mahjoub

**Affiliations:** a Krankenanstalt Rudolfstiftung, Messerli Institute, Veterinary University of Vienna, Vienna, Austria; b University of Tunis El Manar, Genomics Platform, Pasteur Institute of Tunis, Tunisia

**Keywords:** Mitochondrial, Phenotype, Genotype, Encephalopathy, Epilepsy, Lactic acidosis, Complex-I deficiency

Letter to the Editor

With interest we read the article by Apatean et al. about a female infant with a fatal mitochondrial disorder (MID) due to the variant c.612_615dupAGTC in the *FOXRED1* gene [1]. The study raises concerns.

Missing is the family history. We should be informed if the *FOXRED1* variant was transmitted or occurred spontaneously, If any other first degree relative presented with clinical features of a MID, and how the *FOXRED1* variant segregated across the generations.

The index patient developed seizures on the second day of life and received phenobarbital (PB) [1]. Since PB can be mitochondrion-toxic [2], we should be informed in which dosage and for how long PB was given, if PB stopped seizures, or if seizure-frequency declined. It should be also mentioned if PB exhibited any side effects and if deterioration of the phenotype was attributable to the application of PB.

The authors attributed pulmonary hypertension (PH) to the mitochondrial defect [1]. However, echocardiography on the second day of life revealed patent ductus arteriosus and patent foramen ovale [1]. Thus, it should be discussed if PH was simply due to these shunts, which may have increased pulmonary artery pressure, and after a certain point resulted in the reversal of the left-right shunt into a right-left shunt. On follow-up echocardiography 2.5 m after birth the patent ductus arteriosus was no longer visible but nothing is reported about the foramen ovale.

Concerning the application of dichloroacetic acid (DCA) it is well known that it is neurotoxic in MID patients [3] and may cause hepatomegaly and reduced activity in pediatric MIDs [4]. Thus, we should be informed about the rationale for providing this toxic compound.

Overall, this interesting report could be more meaningful if the family history was provided, if first-degree relatives were genetically investigated, and if application of DCA and PB and the pathogenesis of PH were extensively discussed.

## Conflict of interest

There are no conflicts of interest.

## Funding

No funding was received.

## Author contribution

JF: design, literature search, discussion, first draft, SZ-M: literature review, critical comments, revision of first draft.
